# Immunotherapy and immunochemotherapy in combating visceral leishmaniasis

**DOI:** 10.3389/fmed.2023.1096458

**Published:** 2023-05-17

**Authors:** Ganesh Yadagiri, Aakriti Singh, Kanika Arora, Shyam Lal Mudavath

**Affiliations:** ^1^Infectious Disease Biology Laboratory, Chemical Biology Unit, Institute of Nano Science and Technology, Mohali, Punjab, India; ^2^Department of Veterinary Biosciences, The Ohio State University, Columbus, OH, United States

**Keywords:** visceral leishmaniasis, chemotherapy, drug resistance, immunotherapy, immunochemotherapy

## Abstract

Visceral leishmaniasis (VL), a vector-borne disease, is caused by an obligate intramacrophage, kinetoplastid protozoan parasite of the genus *Leishmania*. Globally, VL is construed of diversity and complexity concerned with high fatality in tropics, subtropics, and Mediterranean regions with ~50,000–90,000 new cases annually. Factors such as the unavailability of licensed vaccine(s), insubstantial measures to control vectors, and unrestrained surge of drug-resistant parasites and HIV-VL co-infections lead to difficulty in VL treatment and control. Furthermore, VL treatment, which encompasses several problems including limited efficacy, emanation of drug-resistant parasites, exorbitant therapy, and exigency of hospitalization until the completion of treatment, further exacerbates disease severity. Therefore, there is an urgent need for the development of safe and efficacious therapies to control and eliminate this devastating disease. In such a scenario, biotherapy/immunotherapy against VL can become an alternative strategy with limited side effects and no or nominal chance of drug resistance. An extensive understanding of pathogenesis and immunological events that ensue during VL infection is vital for the development of immunotherapeutic strategies against VL. Immunotherapy alone or in combination with standard anti-leishmanial chemotherapeutic agents (immunochemotherapy) has shown better therapeutic outcomes in preclinical studies. This review extensively addresses VL treatment with an emphasis on immunotherapy or immunochemotherapeutic strategies to improve therapeutic outcomes as an alternative to conventional chemotherapy.

## Introduction

Leishmaniasis is one of the seven most important tropical diseases representing a serious world health problem on a broad spectrum of clinical manifestations with a potentially fatal outcome. It is a vector-borne chronic infectious disease attributable to an intracellular one-celled protozoan parasite of the genus *Leishmania*. It is systemically transmitted to humans by the bite of an infected female *Phlebotomine* sand fly (1). Its incidence has increased to more than 12 million people in 98 countries around the world, mostly afflicting developing and under developed countries (higher in rural areas than in urban areas) (2). The epidemiological burden also depends on the characteristics of the parasite, ecological characteristics of the transmission site, and the immune response of the host. In accordance with the World Health Organization (WHO), an estimated 70,000–100,000 new leishmaniasis cases are reported globally, and over 20,000–30,000 deaths occur annually (3). A hemoflagellate vector, a female sand fly of genus *Phlebotomus* in the Old World and *Lutzomyia* in the New World, is responsible for the transmission of this disease to humans (4). Leishmaniasis has gained more public consideration owing to the higher incidence of morbidity; “the London declaration community” on Neglected Tropical Diseases declared to eliminate leishmaniasis as a public health issue by 2020 (5). Discrete species of *Leishmania* cause diverse clinical manifestations ranging in severity from self-healing localized cutaneous ulcers to fatal multi-organ disease. It broadly manifests as cutaneous leishmaniasis (CL), mucocutaneous leishmaniasis (MCL), and visceral leishmaniasis (VL). The outcome of the disease is unwavering by the interplay of parasite characteristics, vector biology, and host factors, with immune responses (6). VL or kala-azar, as it is called in its advanced stages, is a severe chronic systemic infection caused by *Leishmania donovani* (subtropics of South Asia and East Africa; anthroponotic mode of transmission) and *Leishmania infantum* (Europe, North Africa, and Latin America; zoo anthroponotic mode of transmission) (7). VL is the most severe clinical form of leishmaniasis characterized by hepatosplenomegaly, high fever, pancytopenia, and hypergammaglobulinemia, and if left untreated, the disease may worsen over time to severe progressive cachexia, multi-organ damage, secondary infections, and ultimately causes death of the patient (8). VL is ranked second in mortality and fourth in morbidity among tropical parasitic diseases, with 20,000 to 40,000 deaths annually and over 2,000,000 disability-adjusted life years (DALYs) lost (6). In conformity with the WHO, perennially 50,000–90,000 new VL cases arise globally, and >95% of new VL cases are reported in 10 countries: Brazil, China, Ethiopia, India, Iraq, Kenya, Nepal, Somalia, South Sudan, and Sudan (3). More characteristically, a conclusive 80% of the global burden of VL was reported in South Asia (e.g., in the year, 2007, 0.1–0.15 million VL cases were reported in India alone). The condition is particularly severe in eastern states of India, including Bihar, Jharkhand, Uttar Pradesh, and West Bengal (9). A total of 6,70,897 VL cases were reported officially from 1987 to 2011 from Bihar state exclusively. Some districts of Bihar state (Muzaffarpur, Purnea, Saharsa, Ararea, Vaishali, Madhepura, East Champaran, Samastipur, Saran, and Darbhanga) have confronted worst epidemic since the 1970s with 90% of the case reports (10). Collectively, an integrated elimination program was started by the government of Bangladesh, India, and Nepal started in 2005 with the aim to decline the VL case reports to less than one new case per 10,000 population per year at sub-district level (block level in India and Nepal and upazila level in Bangladesh) by 2015 (11).

The treatment and control of VL are limited and rely mainly on chemotherapy. Existing anti-leishmanial chemotherapeutic agents (meglumine antimoniate, sodium stibogluconate, pentamidine, amphotericin B, miltefosine, and liposomal amphotericin B) encompasses several problems with regard to safety and efficacy. Nephrotoxicity, pancreatitis, cardiotoxicity, teratogenicity, emergence of drug-resistant parasites, high cost, and exigency for hospitalization due to longevous intravenous treatment present challenges toward patient compliance (12). Hitherto, several clinical trials have been performed in India to improve the therapeutic regimens and to ameliorate the efficacy of the limited armamentarium of existing anti-leishmanials (11). The limited therapeutic efficacy of human vaccine(s) and inefficient vector control measures impose perplexity in the treatment of VL. Moreover, leishmania–HIV (human immunodeficiency virus) coinfected people have accelerated chances of developing a full-blown disease with high relapse and lethality. Such cases of co-infections are particularly reported in Western Europe, India, Brazil, Ethiopia, and Africa. These co-infections lead to diagnostic difficulty and therapeutic unresponsiveness to VL treatment (13). Post-kala-azar dermal leishmaniasis (PKDL) is a neglected complication of VL, playing a significant role in the inter-epidemic period as a potential reservoir for the *Leishmania* parasite. It usually appears as a macular, maculopapular, or nodular rash on the face, upper arms, trunks, and bared parts of the body. PKDL is a deadly, infectious disease that is reported in Africa particularly in Sudan (50%; *L. infantum*), and in Asia particularly in India (5–10%; *L. donovani*) (14). The emergence of drug-resistant strains of the *Leishmania* parasite, severe toxic effects of current anti-leishmanial therapy, advent of HIV-VL co-infections, PKDL, and absence of proper vector control measures and vaccine(s) against VL pose severe problems to VL treatment and control (15). Therefore, prevention and control of leishmaniasis require a combination of intervention strategies that would begin with the search for a novel system to actualize the treatment shorter, safer, efficacious, and more affordable (16). Drug discovery for such a poverty-ridden neglected tropical disease was never included as a high priority by pharmaceutical companies as it is unlikely to yield a good profit on research and drug development costs.

In this extant, we have extensively addressed the leading-edge and provocation in therapeutics and ministrations of VL with emphasis on immunotherapy or immunochemotherapeutic strategies to improve therapeutic outcomes as an alternative to prevalent chemotherapy.

### Immunobiology of VL: cells and immune mediators correlated to susceptibility and resistance

**Figure 1:**
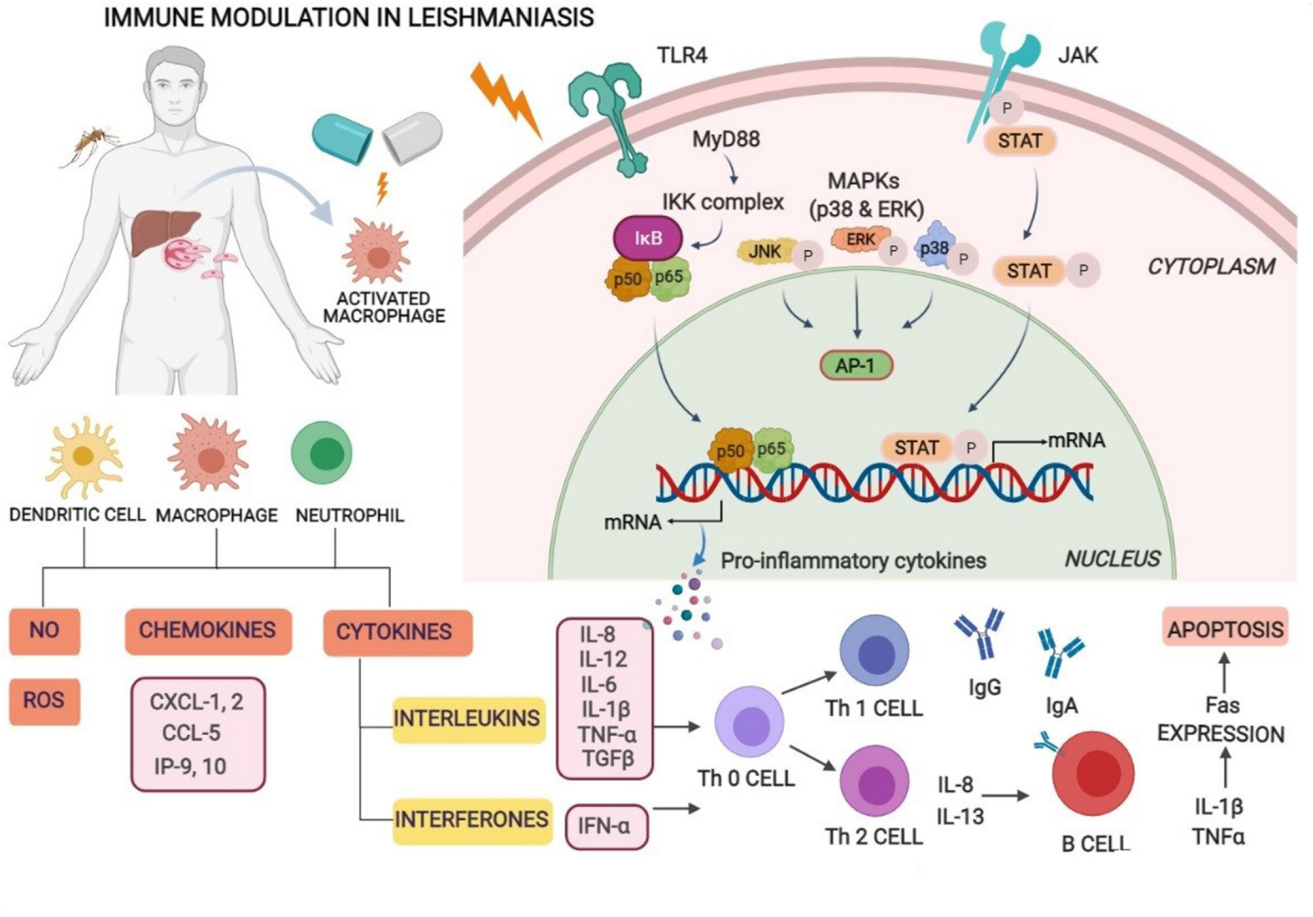
Immune modulation in visceral leishmaniasis. Macrophages, neutrophils, natural killer (NK) cells, and dendritic cells (DCs) play an essential role by sensing parasitic first occurrence *via* pattern recognition receptors (PRRs) and complement receptors present on host cells. Recognition and interaction stimulate intracellular signaling pathways that lead to the occurrence of inflammatory responses and control parasite multiplication. *Leishmania*-infected macrophages and dendritic cells activate Th1 cell differentiation and generate inflammatory cytokines especially IL-12, IFN-γ, reactive oxygen, and nitric oxide species supporting intra-cellular parasite clearance and enhanced levels of Th2 cytokines resulting in disease progression.

The immunobiology and immunopathology of visceral infection for various species including humans, canines, and experimental small animal (rodent) models have been comprehensively studied, with many points exemplified but yet some to be explicated. General consent is that despite the uniqueness of each model, the infection extent and spread are basically influenced by many factors such as concomitant pathologies, pathogen–host interaction, geographic location, and host immune response (17). Other factors, for instance, genetic differences of the parasite between species and strains, host genetics, and environmental factors (18), also provocate the susceptibility or resistance to infection. Parasites have hence evolved to evade and evert host immune responses. Different types of immune cells such as macrophages, neutrophils, natural killer (NK) cells, and dendritic cells (DCs) play an essential role by sensing parasitic first occurrence *via* pattern recognition receptors (PRRs) and complement receptors present on host cells. Numerous toll-like receptors (TLRs) such as TLR2, TLR3, TLR4, TLR7, and TLR9 similarly contribute to the sensing and recognition of *Leishmania* parasites. Recognition and interaction stimulate intracellular signaling pathways that lead to the occurrence of inflammatory responses and control parasite multiplication *via* an innate immune response (19). The macrophages and DCs are known to play crucial roles in the initiation, expansion, and maintenance of an assertive immunity against *Leishmania* infection. Nonetheless, the intramacrophage amastigotes, once internalized, alter cell-signaling pathways in macrophages inhibiting cytokine responses, and this disrupts the protective capacity of cells. These work by inhibiting MAP kinases, transactivation of NF-κB by local stimulation of TGFβ32, and suppression of cytokine signaling (SOCS-3) (20). VL-infected patients evince an accretion of dysfunctional T cells due to their incapability to produce a cell-mediated immune response in defiance of inchoate antigens. This leads to immunocompromisation of VL patients and is prone to secondary pathogenic infections (21). Cellular immunity rather than humoral mediated immunity plays a crucial role in the control of *Leishmania* infection. The progression of type-I immune responses against leishmania is characterized by antigen-presenting cells (APCs) that stimulate the production of interleukin-12 (IL-12) which in turn facilitates interferon-γ (IFN-γ) *via* Th1 T cells. Consequently, it activates macrophage-mediated microbicidal mechanisms which function by producing nitric oxide (NO) and reactive oxygen species (ROS) which efficaciously kill intra-macrophage amastigotes. An immunosuppressive cytokine, IL-10, deactivates the detectable levels of Th1 cytokines which might result in the progression of VL regardless of immune functionality (22). The coaction of Th1 and Th2 cytokines seems to persist throughout the infectious phase which advocates significant roles in disease protection and pathogenesis by various cytokines (23). The resistance and susceptibility of infection depend on the host's immunological status. Enhanced levels of different cytokines of CD4 T-helper cells such as IFN-γ, IL-12, and IL-2 with a lack of IL-10 is requisite for the revivification of VL infection (24). Therefore, enhanced levels of Th2 cytokines result in disease progression while that of Th1 cytokines arbitrates disease clearance (1). Specifically, in BALB/c mice, the replication of amastigotes in the liver marks the first week of infection. Genetically resistant mice (C57BL/6) have natural resistance-associated macrophage protein 1 (NRAMP 1) gene (Slc11a1) mainly exhibited on macrophage surface and involved in macrophage activation that might function by killing the *Leishmania* parasite by nitric oxide (NO)-mediated mechanisms (25). It mainly elevates the expression of IFN-γ by CD4^+^ T cells and exacerbates the disease situation with the scarceness of IFN-γ levels in *Leishmania*-infected C57BL/6 (resistant) and BALB/c (susceptible) mouse strains, respectively (26). A balanced proportion of the cytokine release from the CD4^+^ and CD8^+^ T- ells is a critical factor of immune function against leishmaniasis infection. In active VL, both CD4 and CD8 cells function cooperatively for clearance of infection displaying diverse functions and expression of the cytokines. CD4^+^ cells are critical for the deterrence of primary infection, whereas CD8^+^ cells are role players during the secondary immune response (9). Over expression of chemokines, chemokine (C-X-C motif) ligand 9 [CXCL9], and CXCL10 in serum during active infection counseled that these chemokines accompanied by IFN-γ play a crucial role in the immunopathogenesis of VL (23) (Figure 1).

However, the unification of innate and acquired immunity together with the insufficiency of data on the human immune response is one of the crucial obstacles currently impeding vaccine development and application.

### Current VL chemotherapy and its limitations

Certain factors accounted for the selection of specific drugs in the primary VL zones and therapeutic options including drug risk-benefit ratio, maintenance of health protection, and the accessibility of anti-leishmanial medication in the view of public wellbeing and the epidemiological aspects of vector, parasite, and domestic reservoir of VL (zooanthroponotic and anthroponotic) (27). In the Indian subcontinent, VL is anthroponotic and a major challenge is widespread resistance to antimonials, particularly in Bihar, which is the hyper endemic zone of VL. In Southern Europe and America, VL is zooanthroponotic, and dogs are reservoirs, and is a serious issue toward the control of the infection (28). Due to the non-availability of an effective vaccine against VL, control of VL exclusively relies on chemotherapy. The therapeutic options available for the treatment of VL are summarized in Table 1.

**Table 1:** WHO recommended treatment regimens for VL, in the Indian subcontinent and its mode of action, advantages, and limitations (11, 29).

**S.No**.	**Treatment**	**Mode of action**	**Advantages and limitations**
1.	Liposomal amphotericin B (AmBisome) *[3–5 mg/kg/day, (i.v.) for 3–5 days (total dose of 15 mg/kg) or 10 mg/kg, (i.v.) as a single dose]*	Target specific drug delivery to macrophages (spleen, liver, and bone)	*Advantage*: Highly efficacious and safe *Limitation*: High cost of therapy
2.	Amphotericin B deoxycholate (AmB) *[0.75–1.0 mg/kg/day, (i.v.) daily or on alternate days for 15–20 doses]*	Interacts with ergosterol, existing in the cell membrane of leishmania parasite	*Advantage*: Highly effective in Indian subcontinent, where antimonial resistance emerged *Limitation*: Hypokalemia, myocarditis and nephrotoxicity
3.	Miltefosine *[2.5 mg/kg/day; (p.o.) for children (2–11 years); for people aged 12 years and <25 kg body weight, 50 mg/day; 25–50 kg body weight, 100 mg/day; > 50 kg body weight, 150 mg/day; for 28 days]*	Alteration of alkyl lipid metabolism and phospholipid biosynthesis of leishmania parasite	*Advantage*: Orally active anti-leishmanial drug *Limitation*: Fetal abnormalities in pregnant women
4.	Paromomycin *[15 mg/kg/day (i.m.) for 21 days]*	Inhibits protein synthesis by interacting 30s ribosomal subunit of parasite	*Advantage:* Efficacious in drug combinations *Limitation*: Nephrotoxicity and ototoxicity
5.	Pentavalent antimonials *[20 mg/kg/day for 30 days (i.m.)/(i.v.)]*	Sb^v^ is converted into toxic Sb^III^ form and induce oxidative stress to intra-cellular amastigotes	*Advantage*: Low cost and easily accessible *Limitation*: Drug resistance has reported in VL endemic areas

#### Pentavalent antimonials

Antimony (Stibium) is a semi-metallic element having atomic number 51 that belongs to the vanadium family (Group VA) of the periodic table and has been used in therapeutics since antiquity. An Indian scientist named Dr. Upendranath Brahmachari for the first time in 1920 actualized an antimony-containing substance, that is, urea stibamine, for VL treatment. Later, sodium stibogluconate (Pentostam) and meglumine antimoniate (Glucantime) as synthetic injectable pentavalent antimonials were introduced as primary therapy for CL and VL treatments. It is commonly documented that pentavalent antimonials (SbV) work as a prodrug that is converted into toxic and active trivalent antimonials (SbIII) (reduced form) in the intracellular environment of macrophages, where amastigotes reside and multiply (30). *Leishmania donovani* DNA topoisomerase-I is inhibited by pentavalent antimonial drugs (31). Daily intramuscular injection (20 mg/kg for 28–30 days) has been used as the standard treatment for VL in most parts of the globe. The most frequent side effects of pentavalent antimonials are cardiovascular issues (QTc interval prolongation, ventricular tachycardia, ventricular fibrillation, and torsades de pointes) amongst which QTc interval prolongation (> 0.5 s) signals may lead to the onset of severe and fatal cardiac arrhythmias, arthralgia, pancreatitis, and nephrotoxicity. Pancreatitis is more common with antimonials in HIV-VL co-infected patients and further increases the mortality rate (32). Antimonials (Pentostam or Glucantime) are no longer recommended for clinical use for Indian VL patients due to the rampant rise of drug-resistant parasites. Initially, in 1980s, pentavalent antimonials were administered at a much lower dosage (10 mg/kg) for a short span of 6–10 days, which showed resistance in Indian KA patients. However, in the later years, as reported by Mohapatra (33), the high dosage of 20 mg/kg for a longer duration showed no success in treatment.

#### Amphotericin B

Amphotericin B deoxycholate (AmB), a polyene class of anti-fungal antibiotic has been recommended as a first-line drug for the treatment of VL in the Indian subcontinent for more than four decades. AmB has shown high therapeutic efficiency in Indian KA patients, especially in North Bihar, a VL hyper endemic region (34). Ergosterol, a component in the Leishmanial parasitic cell membrane, is the primary target of AmB. It can bind and sequester ergosterol, leading to the formation of pores in the cell membrane of the parasite (35). AmB has shown improved therapeutic efficiency (~100%) at the dose of 0.75–1 mg/kg, *i.v.*, infusions for 15–20 doses every day or on alternate days; however, most of the KA-infected patients experience infusion-related problems such as fever, thrombophlebitis, chills, hypokalaemia, nephrotoxicity, and myocarditis. These adverse effects require continuous monitoring and hospitalization, thereby increasing the cost of therapy (36). Lipid-based carrier systems for AmB delivery have been developed to overcome the toxic adverse effects of conventional AmB. Liposomal AmB (AmBisome) was introduced with a unique safety profile. Single dose AmBisome (10 mg/kg) was found to be adequate to efficaciously treat VL with an improved safety profile and has now been recommended as a drug of choice to treat VL in the Indian subcontinent (37, 38). As per the recommendations of the WHO's expert committee on the control of leishmaniasis, AmBisome was declared as a first-line treatment drug for VL in India, Bangladesh, and Nepal (39).

#### Pentamidine

Pentamidine, chemically known as 4-[5-4-carbamimidoylphenoxy] pentoxyl benzenecarboximidamide, is an orphan drug approved for the treatment of *Pneumocystis carinii*, a serious fungal opportunistic infection in immunocompromised patients in the United States (40). The anti-protozoal effects of pentamidine isethionate, an aromatic diamidine, were reported against *Trypanosoma cruzi* in 1938. Soon after, reports of antimonial drug resistance, pentamidine, were recommended as a second-line anti-leishmanial drug in KA-infected patients (41). Pentamidine isethionate is recommended as secondary prophylaxis in addition to anti-retroviral drugs to inhibit relapse in HIV-VL co-infected immunocompromised patients (42). Pentamidine selectively binds to kinetoplastid DNA (kDNA) of *Leishmania donovani* resulting in the inhibition of parasite replication (43). Pain or formation of abscess at the site of injection, renal insufficiency, and allergic reactions, including Stevens–Johnson syndrome, cardiotoxicity, and metabolic disorders, are the common adverse effects of intravenous pentamidine isethionate injections (44).

#### Paromomycin

Paromomycin (formerly known as aminosidine) is an aminoglycoside antibiotic with a unique spectrum of anti-leishmanial activity. Paromomycin interacts with the ribosomes of mitochondria and induces respiratory dysfunction in *Leishmania* parasites. Paromomycin binds to the 30S subunit of ribosomes which causes the inhibition of protein synthesis (45). Paromomycin (11 mg/kg/day for 3 weeks, *i.m*. route) was recommended as a first-line treatment for VL in the Indian subcontinent (46–48). However, paromomycin was found to effect the renal (nephrotoxicity), vestibular, and auditory organs (ototoxicity) in VL-infected patients (49).

#### Miltefosine

Miltefosine (hexadecylphosphocholine, HePC), an alkyl phospholipid compound, is the first approved oral therapeutic agent registered for combating VL. Initially, it was used as an anti-neoplastic agent originally designed for breast cancer and other solid tumors. Miltefosine was licensed as an oral anti-leishmanial drug in the Indian subcontinent in 2002 (50). Miltefosine functions by disrupting lipid metabolism *via* inhibition of phosphatidylcholine synthesis, thereby affecting cell-signaling pathways and membrane synthesis of the *Leishmania* parasite. Miltefosine mainly interacts with the acidocalcisome and stimulates the sphingosine-dependent plasma membrane calcium channels of the *Leishmania* parasite (51). Miltefosine (2.5 mg/kg/day for 28 days, *p.o*.) has been recommended for CL- and VL-infected patients with good cure rates (52). Miltefosine is not recommended for pregnant women due to its teratogenicity (fetal abnormalities) (50).

#### Sitamaquine

Sitamaquine (WR-6026) is an orally active 8-aminoquinoline analog developed by Walter Reed Army Institute Research in collaboration with GlaxoSmithKline. Sitamaquine has shown improved efficacy against *L. donovani*-infected patients in Kenya and the Indian subcontinent in phase 2B clinical trials (53). Sitamaquine acts by entering the parasite through diffusion in a sterol-dependent and energy-independent electrical gradient manner (54). Renal toxicity and methemoglobinemia are very common side effects with higher doses of sitamaquine in VL-infected patients. Furthermore, the decision of its clinical development is hindered because of its safety concerns (55).

WHO recommended combination treatment regimens for VL, in the Indian subcontinent (29).

1. Combination of AmBisome (5 mg/kg (*i.v*.) as a single dose) + Miltefosine (2.5 mg/kg/day; (*p.o*.) for children (2–11 years); for people aged >12 years and <25 kg body weight, 50 mg/day; 25–50 kg body weight, 100 mg/day; > 50 kg body weight, 150 mg/day) for 7 days.
2. Combination of AmBisome (5 mg/kg (*i.v*.) as a single dose) + Paromomycin (15 mg/kg/day (*i.m*.)) for 10 days.
3. Combination of Miltefosine (2.5 mg/kg/day; (*p.o*.) for children (2–11 years); for people aged >12 years and <25 kg body weight, 50 mg/day; 25–50 kg body weight, 100 mg/day; > 50 kg body weight, 150 mg/day) for 10 days + Paromomycin 15 mg/kg/day (*i.m*.) for 10 days.

### Immunotherapy and immunochemotherapy: promising strategies for VL treatment

**Figure 2:**
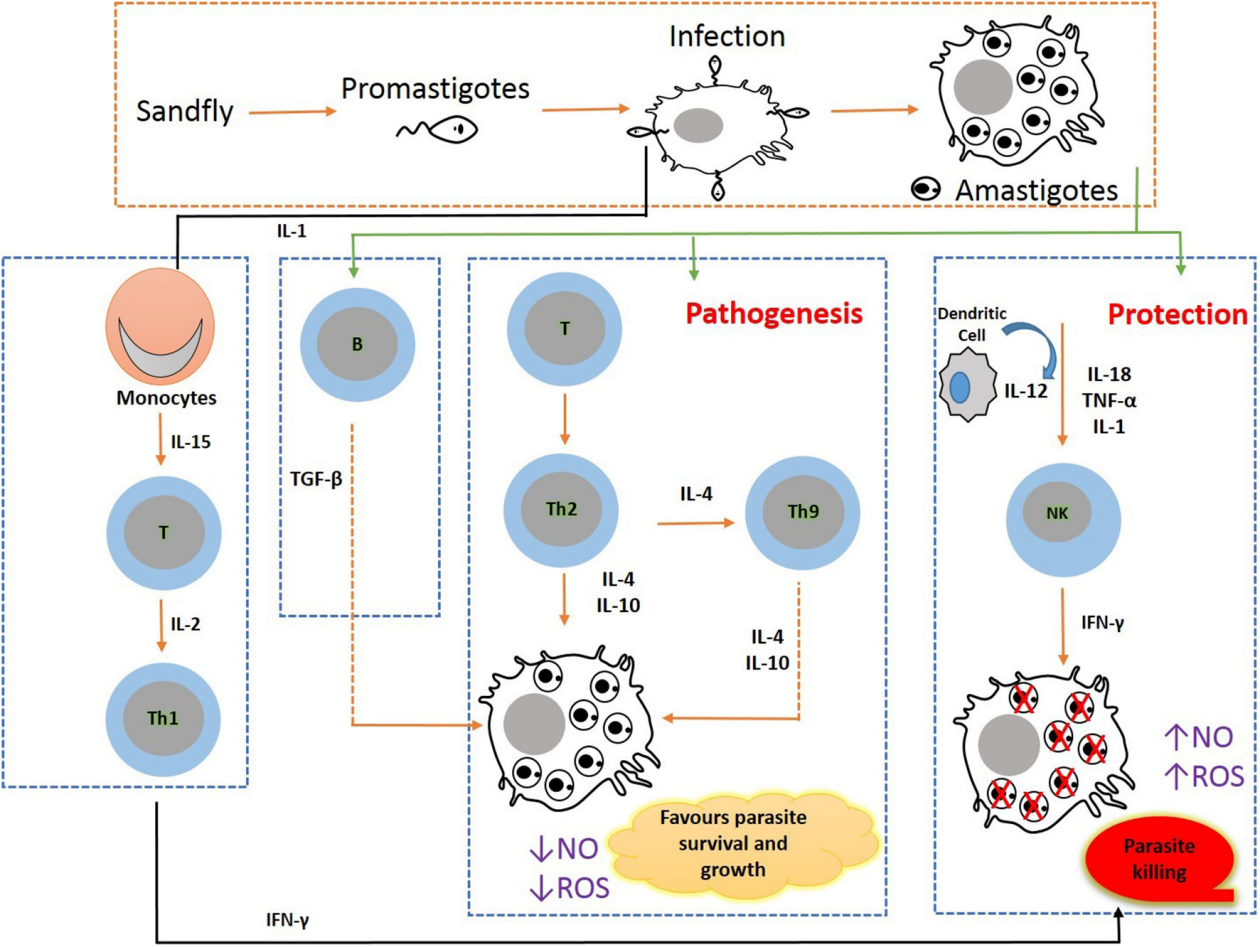
Immunobiology during pathogenesis in visceral leishmaniasis: Leishmania parasite invades macrophages and activates CD4 T cells to release Th1- (IL-12, IFN-γ, and nitric oxide) and Th2 (IL-10 and TGF-β)-type cytokine responses. VL pathogenesis is mediated through the augmented expression of Th2-type cytokines, and VL clearance is arbitrated through the increased Th1 cytokine responses.

Efficient chemotherapy is the alternative for the treatment of VL due to inefficacious vector preventive techniques available for humans so far. Nonetheless, the emergence of drug-resistant parasite strains, drug unresponsiveness, discrepancy in medical responses due to geographical distribution, and disease relapse to the conventional anti-leishmanial therapeutic strategies enforce the concerted efforts for the search of developing new therapies and treatment regimens which can be given orally (56). In such a situation, immunotherapy alone or in combination with conventional chemotherapy can become a viable alternative in the treatment and control of VL. The molecular pathways that lead to the development of parasites are inhibited using biological response modifiers to prevent leishmanial-associated immunosuppression. Thus, this immunotherapy approach has proven its novelty offering advantages of nominal toxicity and cost-effectiveness that could further ameliorate disease progression (9). To attain prophylactic and/or therapeutic success, a biological molecule that has a therapeutic potential to modulate the immunological status of the host is the need of the hour. Presently, immunotherapy strategies have been employed for the successful treatment of several diseases including cancers, allergy-mediated diseases, and some viral infections (hepatitis) (19). Immunotherapy in combination with conventional chemotherapy displays rapid parasite clearance, thereby augmenting the efficacy of treatment (57). Immunotherapy can clear intracellular amastigotes by modulating immunological mechanisms involving the activation of macrophages and cell-mediated immune responses in VL infection (58). For hindering VL infections, Th1-type cytokine responses are to be selectively induced. This approach could successfully augment molecular interactions of immune cells and released cytokines, such as Th1 cytokines, IFN-gamma, and IL-12 levels, and downregulate IL-10 and IL-14, thereby preventing the enhancement of disease severity leading to treatment and simultaneously offering a unique diagnostic approach for VL (59). VL developed as a substantial opportunistic infection in immunocompromised patients with HIV infection (HIV-VL co-infections) in African regions, the Indian subcontinent, and Western Europe and has also shown unresponsiveness to conventional chemotherapeutics. In HIV-VL co-infections, the treatment outcome with monotherapy is poor (60). The conventional approach of anti-leishmanial chemotherapy can be defeasible subject to a high degree of infection and therapeutic situation. To overcome such a stage, the incorporation of an immunomodulator with prevailing chemotherapies can be promising as this combination will stimulate cell-mediated immune response and can result in successful outcomes (Figure 2).

The host immunological state in VL is a key factor that significantly affects infection susceptibility and resistance. The Th1-type cytokines in the CD4 T-helper cells are increased and activated by the microbicidal process mediated by macrophages. As a result, nitric oxide (NO) and reactive oxygen species (ROS) are produced, both of which are extremely efficient for eliminating intramacrophage amastigotes. The increased levels of Th2-type cytokines that can deactivate macrophages to prevent the generation of NO and ROS boost amastigotes' intracellular development.

The neutrophils, macrophages, dendritic cells, and natural killer cells work together to mediate the innate immune response in leishmaniasis. The female sand fly takes a blood meal to start the life cycle of the *Leishmania* parasite. The metacyclic promastigotes subsequently enter the bloodstream and are phagocytosed by neutrophils at the infection site. A greater number of neutrophils migrate to the infection site as a result of the neutrophils phagocytosing the parasite and secreting IL-8. Chemotaxis of macrophages to the infection site is caused by the apoptosis of infected neutrophils and the subsequent release of the macrophage chemokine MIP-1. The parasites' binding to C3b speeds up phagocytosis, and promastigotes are transformed into the amastigote form. The phagocytosed parasites can survive in apoptotic neutrophils and grow in macrophages, causing the spread of the disease. To induce the Th1 response necessary for parasite eradication in infected parasites, *Leishmania* parasites block the production of IL-12 in macrophages, which promotes parasite proliferation. Dendritic cells are prevented from presenting the T lymphocytes with the parasite-specific antigens by the suppression of interleukin (IL)-12 secretions. By enhancing the production of arginase, which inhibits NO generation, *Leishmania* parasites also promote the activation of ornithine from arginine. The conventionally activated macrophages produce pro-inflammatory microbicidal and tissue-damaging cytokines, while the alternatively activated macrophages are anti-inflammatory and regulate inflammation and tissue repair. The signals produced by the microenvironment are necessary for macrophage polarization into M1 and M2. LPS, IFN, TNF, and GM-CSF are the main causes of M1 macrophage polarization, which also activates the complement system and draws in immune cells. M1 macrophages can differentiate into M2 and vice versa, M2 macrophages repolarize more quickly than M1 macrophages, and M1 macrophages can develop into M2 and vice versa. By polarizing macrophages into the M2 subset and reducing dendritic cells, the *Leishmania* parasite impairs antigen presentation and establishes an environment that is immunosuppressive to sustain its survival (61). Furthermore, chemokines including MIP-1, cytokines, and complement proteins induce monocytes to the site of infection, facilitating parasite replication. IL-12, which is essential for the development of CD4+ T cells into protective Th1 cells for the expression of co-stimulatory molecules CD40, CD80, and CD86, is primarily produced by dendritic cells as the disease progresses. A higher parasite load results from blocking CD40–CD40L interaction because it reduces IL-12 and IFN-γ productions. In addition, NK cell suppression reduced IFN production and markedly increased the parasite load (13, 62). Numerous studies have shown that immunotherapy is a successful anti-leishmanial treatment. Activation of macrophages was increased by a single dose of an IL-27 or IL-10 monoclonal antibody, which improved parasite killing (56%). The synthesis of the anti-inflammatory IL-17 is adversely regulated by IL-27 inhibition, which worsens the condition (63). It has been discovered that IFN-induced macrophage killing of *Leishmania* is inhibited by the macrophage deactivation cytokines IL-4 and IL-10, which promote the development of VL. Additionally, TGF antibody neutralization increases IFN-γ production, which in turn increases IL-2-associated macrophage activation, nitric oxide generation, and proliferation of cytotoxic T lymphocytes, curing VL independently of TH2-type cytokines (IL-4). Additionally, IL-10 inhibition promotes VL regression (64). A pleiotropic cytokine called IL-6 regulates dendritic cell activity during infection with *L. donovani*. IFN-γ and tumor necrosis factor can be used to activate macrophages, but IL-6 directly impairs their ability to do so. It also promotes the repression of Th2-type responses and inhibits the development of Th1 cells. An achievable therapeutic target is the immunosuppressive, macrophage-deactivating nature. *L. donovani* infection in the liver has been linked to cytokine antagonist therapy with IL-6, IL-10, and IL-27. In contrast, IL-10, IL-6, and IL-27 receptor signaling directly affects *L. donovani* liver infection and, when absent, enhances Th1-type responses, speeding up parasite elimination (65). As inflammation reaches a particular level, the cytokine IL-22, a member of the IL-10 family, causes non-immune cells such as epithelial cells and fibroblasts to proliferate and migrate, aiding in tissue protection. Strong IL-17 and IL-22 induction by *L. donovani* leads to additional tissue healing that aids in immunosurveillance and suggests their complementary roles in host defense against the parasite (66). The immunotherapy and immunochemotherapeutic approaches against visceral leishmaniasis is highlighted in Table 2.

**Table 2:** Immunotherapy and immunochemotherapeutic approaches against visceral leishmaniasis.

**S.No**	**Immunotherapeutic agent/immunomodulators**	**Chemotherapeutic agent**	**Efficiency/clinical outcomes**
1.	IFN-γ (10^7^ U/mg, once daily/30 days; *s.c*.)	Sodium stibogluconate (20 mg/kg/day for 30 days)	87% cure rate in Human VL; IFN-γ immunotherapy shortens the duration of conventional chemotherapy (67)
2.	IFN-γ (1.9 × 10^7^ U/mg; *i.p*.)	Mannosylated liposomes encapsulating doxorubicin B	Safe and effectively cures murine VL with augmented Th1 cytokine responses (68)
3.	LeishF3+GLA-SE	-	Safe and elicited a robust antigen-specific CD4 Th1 immune responses in human and murine VL (69)
4.	rh GM-CSF (150 mcg; *s.c*. twice weekly for 12 consecutive weeks)	Amphotericin B (4 mg/kg per day, *i.v*.)	Safe and efficacious in HIV-VL co-infected patients (70)
5.	rh GM-CSF (5 g/kg daily for 10 days, *s.c*.)	Glucantime (10-20 mg/kg daily for 20 days, *i.v*.)	Reverse neutropenia and reduces secondary infections in human VL (71)
6.	rm IL-12 (2.7 × 10^6^ U/mg)	Amphotericn B (5 mg/kg, *i.v*.)	rm IL-12 potentiates Amphotericin B efficacy in murine VL (72)
7.	rLdT-E (recombinant chimeric triosephosphate isomerase and enolase)	-	Improves cellular and humoral immunity in *L. donovani*-infected hamsters (73)
8.	Octyl-β-D-galactofuranose (Galf)	-	Augments Th1 responses and diminishes parasite burden in murine VL (74)
9.	Picroliv (10 mg/kg, 12 days, *p.o*.)	Ketoconazole (50 mg/kg, 5 days, po) + miltefosine (5 mg/kg, 5 days, po)	82% efficacy and improves cell mediated immunity in *Leishmania-*infected hamsters (75)
10.	*L. braziliensis* antigens + saponin + monophosphoryl lipid-A	Miltefosine	Augmented CD4+ T cells in splenocytes producing IFN-γ and TNF-α and a reduction of IL-10 and anti-Leishmania circulating IgG in hamsters (76)
11.	recombinant cysteine proteinase from *Leishmania*, rldccys1	Miltefosine (46 mg/kg) or allopurinol (460 mg/kg)	Significant decrease of parasite load in infected hamsters (77)

### Cytokine therapy for VL

Small, soluble proteins known as cytokines play a critical role in the control of innate immunity. They are primarily produced by mononuclear phagocytes such as macrophages and dendritic cells (78). Th2-type cytokines are expressed more frequently in VL, which regulates the course of the disease, and Th1 cytokines are produced at higher levels, which intervene to resolve the infection (1). The identification of cytokines that preferentially activate Th1-type cytokine responses may be useful in the treatment of VL. Anti-IL-10 antibodies are being investigated for use in monotherapy (immunotherapy) or in conjunction with conventional anti-leishmanials. Immunostimulatory cytokines (GM-CSF, IFN-γ, and IL-12) as well as antibodies that target suppressive or deactivating cytokines are also being investigated (immunochemotherapy).

#### Granulocyte-macrophage colony-stimulating factor

Granulocyte-macrophage colony-stimulating factor (GM-CSF) is an immuno-regulatory glycoprotein cytokine that stimulates hematopoiesis by inducing the differentiation and proliferation of committed progenitor cells of the myeloid lineage in the bone marrow (79). In addition to playing a crucial role in the control of innate and adaptive immunity, GM-CSF activates macrophages to improve phagocytosis, antigen presentation, chemotaxis, and cell adhesion (80). By using an *in vitro* colony-stimulating factor activity assay, Burgess et al. for the first time extracted and purified GM-CSF from mouse lung conditioned medium, which stimulates the proliferation of granulocytes, macrophages, or both, in bone marrow progenitor cells (81). Infectious disorders such as leishmaniasis, malaria, and tuberculosis have all been successfully treated with immunotherapy using recombinant GM-CSF (1). In VL, GM-CSF causes granulocytopenia to improve as well as blood monocyte mobilization and macrophage activation, both of which may help maintain an infection-fighting immune system. Recombinant mouse GM-CSF (rm GM-CSF) therapy efficiently eliminated the infection in experimental VL, but anti-rm GM-CSF antibody therapy made the visceral infection worse (82). Recombinant human GM-CSF (rh GM-CSF) treatment effectively eliminated intracellular amastigotes in *L. donovani*-infected macrophages in an LPS-independent manner, and the amount of time required to stimulate macrophages to produce leishmanicidal effects is very short (36 h for rh GM-CSF and 48–72 h for rh IFN-γ to activate macrophages) (83). Combining rh GM-CSF and M-CSF with rh IFN-γ is a highly effective way to increase the control of intracellular parasites. In patients with VL infection, immunochemotherapy with rh GM-CSF (5 g/kg daily for 10 days) and Glucantime (10–20 mg/kg daily for 20 days) can reverse neutropenia and decrease subsequent infections (71). Patients with HIV and VL can successfully treat the visceral infection with increased safety using immunotherapy with rh GM-CSF (150 mcg, s.c. route, twice weekly for 12 consecutive weeks) and liposomal amphotericin B (4 mg/kg/day for 5 consecutive days, then on day 10, 17, 14, 31, and 38) (70).

#### Interferon-gamma

Interferon-gamma (IFN-γ) (molecular weight 20–25 kDa) is a sole type II IFN, IFN-γ is a pleiotropic soluble glycoprotein cytokine, crucial for persuading and modulating the innate and adaptive immune response. It induces the host defense mechanism and is hence used in immunotherapy against several pathogenic infectious diseases including malaria, tuberculosis, fungal diseases, leishmaniasis, and toxoplasmosis (84). The integral source for the formulation of IFN-γ is T-lymphocytes (helper T cells, cytotoxic T cells, and natural killer cells) which develop strength for parasitic infections by fortifying macrophages. IFN-γ augments the intra-cellular amastigote-killing impact to secrete Th1-type cytokines by aiding in the development of macrophages. Augmented expression of Th1-type cytokines is embroiled in eliminating the infection in the first place while Th2-type cytokines mediate VL pathology (85). For instance, treatment with *L. donovani* antigens (triosephosphate isomerase and enolase) clears the liver and splenic parasite burden with enhanced IFN-γ levels in Syrian golden hamsters (73). CGD4+ T cells were found to be critical for and the key basis of the IFNγ generation in *Leishmania* parasite-stimulated whole blood (WB) cultures. Endogenously generated IFN-γ in active VL patients helps in the control of intracellular parasite growth, and the inhibition of IFN-γ secretion in *ex-vivo* splenic aspirate cultures exacerbates the disease progression (86). CD4+ T cells stimulate the formation of augmented levels of IFN-γ which not only assists in controlling the *Leishmania* parasite but also deteriorates the disease condition by creating a deficiency of IFN-γ in *L. major*-infected resistant (C57BL/6) and susceptible (BALB/c) mouse strains, respectively (26). Immunotherapy with IFN-γ alone has shown anti-leishmanial protective effects in Indian VL-infected patients. In a pilot study, for 20 days, 9 VL-infected patients were treated with rh IFN-γ where patients showed diminished parasites in their splenic aspirates indicating the therapeutic efficiency of IFN-γ (87). Immunochemotherapy with rh IFN-γ in addition to pentavalent antimonials treated the formerly untreated Indian VL patients and proved to be beneficial in minimizing the length of standard chemotherapy (67). Immuno-nano-chemotherapy with rm IFN-γ and mannosylated liposome-incorporated doxorubicin effectively cures the experiment murine VL and this synergetic combined therapy clears the intracellular parasites by inducing nitric oxide synthase, modulates the T-cell expressions from a Th2 to Th1 pattern, and reveals long-lasting resistance (68).

#### Interleukin-12

Interleukin-12 (IL-12) is a heterodimeric pluripotent cytokine with a molecular weight of 70 kDa, comprising two subunits namely IL-12p35 (35 kDa) and IL-12p40 (40 kDa) linked by a covalent bond. Antigen-presenting cells (APCs) such as monocytes macrophages, neutrophils, and dendritic cells are the main source for the generation of IL-12. IFN-γ production, the generation of Th1-type cytokines, the activation of natural killer (NK) cells, and the differentiation of naive CD4+ cells from Th1 cells are all influenced by IL-12 (88). Immunotherapy of infectious diseases congruous with IL-12 can be considered where a Th1 response is necessary. IFN-γ and Th1-type cytokines with much more improvisation would help in controlling (bacterial, viral, or protozoan) pathogenic infectious diseases (89). Augmented levels of IL-12 p40 and IFN-γ were expressed, independently in peripheral blood mononuclear cells (PBMC) from treated VL patients in response to *in vitro* activation with *Leishmania* parasites, and treatment with exogenous rh IL-10 abrogates the IL-12 p40 levels. Consequently, treatment of active VL patient PBMC with IL-12 or anti-IL-10 modulates the response toward a Th1-type response with the release of IFN-γ and suggests that IL-12 may play a crucial role in the modulation of cellular immune responses in human VL (90). Immunotherapy with rm IL-12 efficiently reduced the established systemic intracellular infection in experimental murine VL in IFN-γ mediated mechanism (91). In experimental VL, IL-12 initiates control over *Leishmania* infection through the activation of Th1-type cytokine response, stimulation and release of IFN-γ, and formation of granuloma. *L. donovani*-challenged IL-12p35 gene KO mice allowed uncontrolled liver infection which failed to respond to pentavalent antimonials (which requires T cells and IFN-γ activation). Immunochemotherapy using rm IL-12 synergistically to pentavalent antimonial in normal mice was IFN-gamma dependent; nevertheless, IL-12 also augmented the responsiveness to antimonials in IFN-γ KO mice. Therefore, IL-12 plays a key role in regulating the host immune responses in both IFN-γ-dependent and independent manner that improvises the anti-leishmanial activity of antimonials (92). Co-administration of plasmid encoding for both p35 and p40 subunits of IL-12 and leishmanial recombinant open reading frame F (rORFF) protein provide immunity against experimental VL with an augmented proliferative response of splenocytes and ensuing secretion of Th1 cytokine IFN-γ. Interestingly, IL-12 DNA played a crucial role in regulating the humoral-mediated responses toward the IgG2a isotype signifying its use as a viable vaccine adjuvant against VL (93). Immunostimulatory compounds such as Octyl-β-D-galactofuranose (Galf) clear intracellular parasites in *Leishmania*-infected human monocyte-derived macrophages and murine models with augmented IL-12 expression (74).

#### IL-10 receptor blocker as an alternative approach against VL therapeutics

IL-10 is 18 kDa pleiotropic anti-inflammatory cytokine, predominantly generated by alternatively activated macrophages, DCs, natural killer cells, both Th1 and Th2 cells, B cells, CD4^+^CD25^+^ forkhead box protein 3 (Foxp3^+^) Treg cells, and keratinocytes. IFN-γ and IL-2 produced in Th2 cells are majorly inhibited by IL-10 which then effectively inhibits proliferation and cytokine responses in T cells. This also leads to the arbitration of both immunological insensitivity and the suppression of immune reactions (94). Moreover, IL-10 also functions to impede macrophage-facilitated stimulation of T cells which occurs by the decreased expression of class II major histocompatibility complex (MHC) and co-stimulatory molecules on the macrophage surface. This impedes both innate and T-cell-facilitated immunity (95). IL-10 levels markedly augmented in *L. donovani*-infected macrophages by inhibiting the release of nitric oxide, superoxide (O${}_{2}^{-}$), and TNF-α. Treatment with anti-IL-10 monoclonal antibodies reduces intracellular amastigotes. IL-10 favors the intramacrophage survival of the *Leishmania* parasite through selective impairment of Ca^2+^-dependent protein kinase C-mediated signal transduction (96). A peculiar difference could be seen in symptomatic and asymptomatic VL patients where IL-10 levels show enhanced serum secretions in the former cases. IL-10 essentially functions to protect the tissues from a collateral impairment that occurs due to extreme inflammation. In active VL, CD8^+^ T cells could also play a significant function in the progression of the disease as these are associated with elevated IL-10 inception (97). Neutralization of IL-10 cytokine induces enhanced *Leishmania* parasite clearance in splenic aspirates of VL-infected patients with augmented levels of IFN-γ and TNF-α. This brings light toward another approach to target IL-10 in VL immunotherapy (98). Immunotherapy with anti-interleukin (IL)-10 receptor (IL-10R) monoclonal antibody (mAb) reduces the liver parasite burden in *L. donovani-*infected mice through the enhanced expression of IL-12 protein 40, IFN-γ inducible nitric oxide synthase (iNOS) (99). Human clinical trials have been perceived with the combination of AmBisome and anti-IL-10 mAb which anticipates to prompt synergistic effects in VL-infected patients. The threat of drug resistance and conceivably attaining a chemotherapeutic dose-sparing effect may be conquered through the outcomes in better therapeutic efficiency and treatment adherence (9).

#### Dendritic cell-based immunotherapy

Dendritic cells (DC), originally described by Steinman and Cohn, are professional antigen-presenting cells (APCs), that specifically stimulate naive T-cell activation and effector differentiation. These are mainly considered messengers between adaptive and innate immune systems. DC-based immunotherapies have been successfully employed in the treatment of cancers by manipulating the immune system to attain cancer control and, preferably, cure cancer. The flexibility of cancer immunotherapy was first revealed by Coley, who used a mixture of bacterial toxins to treat sarcomas (100). The traditional therapies being inefficient stipulates an alternative productive approach that can combat parasitic infections and can impart protection against immunity. DC manipulation serves the purpose. Earlier reports have shown that DC-based immunotherapy can prompt protection against different infectious pathogenic microbes, including bacteria, virus, and protozoan parasites. DCs sense microbes *via* TLR or C-type lectin receptors (101). Uptake of the *Leishmania* parasite by DCs requires parasite-reactive immunoglobulin (Ig) G and is mediated through FcγRI and FcγRIII, critical for the optimal development of protective immunity against infection (102). DC subsets contribute extensive polarizing effects on T helper cell differentiation and DC subset 1, exerting Th1 polarization by IL-12 secretion and activation of signal transducer and activator of transcription 4 (STAT4). DCs play a vital role in initial anti-leishmanial T-cell response and in promoting differentiation into memory T cells to accomplish long-lasting adaptive immunity (103). DCs are also the critical source of early IL-12 generation following *Leishmania* infection. IL-12 secretion by DC is transient, peaking at 24 h of post-infection and reaching the levels observed in uninfected mice by 72 h. DC-T-cell clusters offer a microenvironment for initial NK cell activation, which releases IFN-γ, through a pathway that is reliant on IL-2 and IL-12, crucial for the development of host-protective T-cell responses against the *Leishmania* parasite (104). DC-SIGN (DC-specific ICAM-3-grabbing non-integrin), a C-type lectin receptor expressed on tissue monocyte-derived DCs bind with distinctive *Leishmania* species, has been shown to encourage parasite survival. Therefore, DC-SIGN receptor can be considered a therapeutic target for VL (105). Dendritic cells (DCs) evolved from bone marrow (BM) or bone marrow-derived dendritic cells (BM-DCs) resulting in increased parasitic activity in association with *L*. *infantum* histone H1 in BALB/c mice. The decrease in the cells produced by 1L 10 and an increase in IFN-γ producing cells (parasite-specific) are noticed. Moreover, the increased value of the ratio IgG2a/IgG1 concludes the presence of immune responses of Th1. These immuno-stimulatory effects of *Leishmania* histone H1 can help in developing a vaccine against VL infections acting as a competitor protein (106). Immunochemotherapy with soluble *L. donovani* Ag (SLDA)-pulsed syngeneic BMDC and pentavalent antimonials successfully treated experimental murine VL (107).

#### Toll-like receptors

Toll-like receptors (TLRs) are trans-membrane proteins expressed as a membrane or cytosolic receptor on monocytes, neutrophils macrophages, dendritic cells, B lymphocytes, and T lymphocytes. The TLR signaling pathway is one of the first defense systems against invasive pathogenic microbes. TLR family consists of 11 members (TLR1–TLR11), with specificity to the innate immunity cells by pathogen-associated molecular patterns (PAMPs) and toll interleukin1 (IL-1) receptor (TIR) of numerous infectious pathogenic microorganisms (108). TLRs play a crucial role in the activation of macrophages and the control of intracellular parasitic infections. During *Leishmania* infection, TLR1, TLR2, TLR3, and TLR4 expressions were upregulated and stimulated tumor necrosis factor alpha (TNF-α) release by human primary macrophages which are vital for the elimination and control of intracellular parasites (109). Prevention of TLR-4 activation by *Leishmania* inhibitor of serine peptidase 2 in murine macrophages favors survival and growth of the *Leishmania* parasite (110).

During *Leishmania* infection, the involvement of TLR9, TLR4, and TLR2 is critical for causing a pro-inflammatory cytokine response. TLR-9 deficient mice have shown more susceptibility to *Leishmania* infection. Miltefosine treatment significantly induces both TLR4 and TLR9 in *L. donovani*-infected macrophages which might account for eliciting a potent antileishmanial pro-inflammatory response with augmented levels of IFN-γ, IL-12, and iNOS2 accompanied by a consequent reduction in IL-10 and TGF-β levels (111). Electrospray encapsulation of resiquimod, polymeric microparticles–TLR agonist, has shown protection in experimental VL (112). Release of IFN-γ, NO, and ROS *via* TLR7/8 agonists stimulated by macrophages may be proved to be helpful in the clearance of parasites. Liposomal formulation of resiquimod (a TLR 7/8 agonist) has been known to significantly reduce the parasitic burden in the liver, spleen, and bone marrow in addition to enhanced levels of IFN-γ in *L. donovani*-infected BALB/c mice (113). TLR-4 agonist (glucopyranosyl lipid A (GLA-SE) and LEISH-F3 showed protection in *L. donovani* and *L. infantum* infected murine models (69).

### Vaccines

The conventional therapeutics for VL are accompanied by severe toxic adverse effects, including drug resistance against parasites and exorbitant cost. Vaccine-based therapies could provide a better alternative to the current therapeutics which may overcome the shortcomings of present-day anti-leishmanials and could be effectively efficacious in eliminating VL. Efforts are ongoing by researchers to find a suitable vaccine (s) to control VL (114). The development of a safe and effective vaccine mainly depends on the identification of antigens and their delivery platforms that could provoke T-cell responses (115). Generally, the vaccine against leishmaniasis can be categorized as first-generation vaccines (whole-killed parasites), second-generation vaccines (recombinant proteins), and third-generation vaccines. The currently available experimental vaccines for the control and elimination of VL are summarized in Table 3.

**Table 3:** Experimental vaccines for the control and elimination of VL.

**S. no**.	**Vaccine**	**Host**	**Efficacy/clinical outcome**
1.	Leishvaccine [*L. amazonensis* promastigotes with BCG as an adjuvant]	Canine	Activates innate and cell mediated immunity. Protective against canine VL and used as prophylaxis (116)
2.	Radio-attenuated vaccine [*L. donovani* promastigotes]	Balb/c mice	Elicits strong Th1 immune responses and diminishes Th2 cytokine responses confers protection to murine VL (117)
3.	Fucose-Mannose Ligand (FML) vaccine [Fucose and mannose containing glycoprotein-enriched portion isolated from *L. donovani* promastigotes and saponin as an adjuvant]	Balb/c mice	Augmented IgG1, IgG2a and IgG2b antibodies, DTH response and decreased serum IL-10 levels (118)
4.	Leishmune [FML fractions in saponin adjuvant]	Canine	Decrease the parasite burden with enhanced CD4+ T-cell response (119)
5.	HASPB and KMP-11 vaccine [Adenoviral vector comprising *L. donovani* antigens, HASPB and Kinetoplastid membrane protein-11]	Balb/c mice	Reduces liver parasite burden and enhanced antigen specific CD4+ and CD8+ T-cell responses (120)
6.	*Leishmania* KMP-11 DNA construct [*L. donovani* KMP-11 DNA construct]	Syrian golden hamster	To elicit immunity in stibogluconate sensitive and resistant strains. KMP-11 can activate CD8+ T cells followed by the release of interferon-γ (121)
7.	Poly (D,L-lactide-coglycolide) nanovaccine [Nanoparticles loaded with soluble *Leishmania* antigens and monophosphoryl lipid A]	Balb/c mice	Protection against murine VL (122)
8.	Liposomal nanovaccine [Nanoparticles encapsulating soluble leishmania antigens (SLA) along with monophosphoryl lipid-trehalose dicorynomycolate (MPL-TDM)]	Balb/c mice	Augmented protection and cellular immune responses in murine VL (123)
9.	Cationic liposomal nanovaccine [Cationic liposomes encapsulating non-coding plasmid DNA bearing immunostimulatory sequences along with leishmanial antigens]	Balb/c mice	Protection against murine VL (124)
10.	PLGA nanovaccine [PLGA nanoparticles co encapsulated with lipophosphoglycan with soluble and autoclaved *leishmania* antigen]	Balb/c mice	Enhance cellular immune responses in murine VL with increased levels of nitric oxide and IFN-γ (125)

#### First-generation vaccine candidates for visceral leishmaniasis

Whole-killed, fractionated antigens, and attenuated *Leishmania* parasites are the three main important components of first-generation vaccines. The first-generation vaccines are simple and easy to create in developing countries at low cost nevertheless, registration of vaccines fabricated from cultured parasites creates a major hurdle (126). For instance, the Leish vaccine comprises promastigotes of the *L. amazonensis* strain with BCG as an adjuvant that elicited excellent protective immunity in the canine VL model and was used for prophylactic treatment. This first-generation vaccine initially activates the innate immune system followed by cell-mediated immunity with the stimulation of a mixed cytokine profile with interferon-γ and IL-4 (116). Furthermore, γ-irradiated (radio-attenuated) *L. donovani* parasites were used as a vaccine candidate for VL. Intramuscular injection of radio-irradiated *L. donovani* parasites (2 doses once in 15 days) to Balb/c mice elicits strong Th1 immune responses and diminishes Th2 cytokine responses conferring protection to VL. This vaccine restores memory T cells which resulted in the clearing of *intra*-cellular amastigotes by phosphoinositide-dependent kinase 1 (PDK1), phosphoinositide 3 kinase (PI3K), and p38 mitogen-activated protein kinase (p38MAPK) signaling pathways leading to increased release of nitric oxide (117). The production of whole-killed vaccines is theoretically simple, less expensive, and helps in the prevention and control of VL endemic zones in middle- and low-income countries. Nevertheless, standardization of vaccine generated from *in vitro* cultured parasites is challenging, and killed parasites does not resemble a clinical infection and has created problems in commercial vaccine development efforts. Safety and stability are the main concerns for first-generation vaccines, which require further investigation (20).

#### Second-generation vaccine candidates for visceral leishmaniasis

Recombinant purified proteins, which are generated through genetic engineering, are named “second generation vaccines”. On contrary, recombinant proteins with adjuvants or expression in heterologous microbial vectors are used as second-generation vaccines against leishmaniasis. Scalable and cost-effective production approaches can be achieved by recombinant technology and implicit a more viable alternative for mass vaccination campaigns; however, stability is a major concern (127). Fucose-Mannose ligand (FML), the fucose and mannose containing glycoprotein-enriched portion isolated from *L. donovani* promastigotes, strongly inhibited both promastigote and amastigote forms of *L. donovani*. Furthermore, FML is a potent immunogen in rodents and rabbits, present on the surface of the parasite throughout their life cycle. Therefore, it is used in the serodiagnosis of human and canine VL (128). Recently, the immunotherapy with FML of *L. donovani* promastigotes formulated with saponin as an adjuvant has shown protection in *L. donovani*-infected BALB/c mice with enhanced IgG1, IgG2a, and IgG2b antibodies, a delayed-type hypersensitivity (DTH) response and decreased serum IL-10 levels (118). The first commercially licensed vaccine, Leishman, comprised FML fractions in saponin adjuvant against canine leishmaniasis. Administration of this vaccine has been shown to decrease the parasite burden and clinical disease of long-term *L. donovani* infection with enhanced CD4+ T-cell response and lowered serum antibody levels (119). A recombinant adenoviral vector comprising *L. donovani* antigens, hydrophilic acylated surface protein B (HASPB), and Kinetoplasmid membrane protein-11 (KMP11), administered in a single dose to the mice previously infected with *L. donovani* considerably decreased liver parasite burden. This novel vaccination strategy resulted in improved DTH with enhanced antigen-specific CD4+ and CD8+ T-cell responses (120). At present, numerous vaccine prospects, for instance, Leish-F1, F2, and F3, are in clinical trials developed by Infectious Disease Research Laboratory against leishmaniasis. Leish-F1, a human recombinant vaccine could confer a specific degree of protective immunity against *Leishmania* infection (116).

#### Third-generation vaccine candidates for visceral leishmaniasis

DNA vaccination is a novel technology, which uses genetically engineered DNA to elicit an immunological response. The immunogenic protein-expressing genes are cloned into a suitable vector and vaccinated to murine models through the *i.d*. (or) *i.m*. route causing strong Th1 immune responses, ensuing in robust cytotoxic T-cell immunity. Several molecules (A2, KMP-11, ORFF, P36LACK, and KMP-11 PPG) delivered through the DNA vaccine platform to preclinical animal models induce strong antibody and cellular immunity (129). *Leishmania* KMP-11 DNA construct has the potential to elicit immunity in stibogluconate-sensitive and resistant strains in the Syrian golden hamster model. KMP-11 can activate CD8+ T cells followed by the release of interferon-γ (121). A recombinant adenoviral vector comprising *L. donovani* antigens, hydrophilic acylated surface protein B (HASPB), and KMP11, administered in a single dose to the mice previously infected with *L. donovani* considerably decreased liver parasite burden. This novel vaccination strategy resulted in improved DTH with enhanced antigen specific CD4+ and CD8+ T-cell responses (120).

#### Nanovaccines for visceral leishmaniasis

Nanoparticle delivery of vaccines can boost humoral and cellular immune responses in the host. Nanoparticles facilitate the improved antigen uptake by immune cells such as dendritic cells macrophages, nasal-associated lymphoid tissue (NALT), and gut-associated lymphoid tissue (GALT), initiating an effective antigen processing and presentation. This also makes a vaccine more effective to target specific immune cell surface receptors to elicit strong protective immune responses. Nanomaterials can help co-deliver antigen and adjuvant in one cargo to target antigen-presenting cells (130, 131). *Leishmania*-infected BALB/c mice when treated with a combination of poly (D,L-lactide-coglycolide) nanoparticles loaded with soluble *Leishmania* antigens in addition to TNFα mimicking peptide or monophosphoryl lipid A actuate the protection against the disease (122). Vaccination of Balb/c mice with liposomal formulations of soluble *Leishmania* antigens (SLA) along with monophosphoryl lipid-trehalose dicorynomycolate (MPL-TDM) showed enhanced protection with strong cellular immune responses (123). Cationic liposomes encapsulating non-coding plasmid DNA bearing immunostimulatory sequences along with leishmanial antigens showed protection in experimental murine VL (124). PLGA nanoparticles co-encapsulated with lipophosphoglycan with soluble and autoclaved leishmania antigen were shown to enhance cellular immune responses in murine VL model with augmented levels of nitric oxide and IFN-γ (125).

### Combination therapy: interplay of immunochemotherapy

Detailed understanding of host immune response aids in the implementation of a synergistic chemoimmunotherapeutic regime which could exacerbate inflammatory responses leading to direct parasiticidal effects. By inducing the factors that downregulate macrophage-derived nitric oxide function and deactivating macrophage-killing effector functions *via* enhancement of IL-10 *Leishmania* parasites, this therapy hijacks the host immunity by impairment of Th1 differentiation. A combinatorial host-directed chemoimmunotherapy where aiming immune responses in coalition with the chemotherapeutic agent could enhance Th1 response and the corresponding anti-leishmanial activity. An additive effect has been observed in combination with chemotherapy with significant improvement in the clinical condition of patients with severe side effects (132). Combinative therapy of IL-12 in association with an anti-CD40 antibody enhanced the effectuality of a substandard dose of AmB against *L. donovani*. Inhibition of cytotoxic T lymphocyte Ag-4 (CTLA-4) which acts as a negative regulator in the T-cell activation mechanism, enhanced the number of cells producing IFN-γ and IL-4 in both spleen as well as liver of *L. donovani*-infected mice. Co-consortium of anti-CD40 and anti-CTLA-4 with antimonials synergistically enhanced antileishmanial activity (133). Furthermore, IL-4 production stimulates dendritic cells to produce IL-12 mediating a beneficial Th1 response while IL-10 modulates dendritic cells' production of essential reactive nitrogen intermediates (134). Previous studies have reported the synergistic effect of anti-IL-10 with antimony substantiating its potential for immunochemotherapy (135). Antimony-based chemotherapy in coalition with dendritic cell-specific immunotherapy potentiated the antileishmanial activity by an additive effect of enhanced IFN-γ signaling and the ability to potentiate the production of reactive oxygen species leading to induction of programmed cell death in *Leishmania* parasite (107).

Currently, enhanced efficacy mainly relies on the action of type-1 and type-2 cytokines (IL-2, IL-4, IL-12, IFN-γ, and TNF-α), activation of reactive oxygen species and nitric oxide (NO) production in macrophages, and CD4^+^ and CD8^+^ T-cell subsets when infected macrophages are co-treated with pentavalent antimonials (136). Additionally, combinatorial therapy of AmB with IL-12 and anti-IL-10 receptor boosts Th1 implications and hence clearance of *L. donovani* (137). Miltefosine upsurges the production of IFN-γ, TNF-α, and IL-12, which increased phagocytosis in macrophages with augmented NO production favoring a protective immune response (134). Miltefosine along with immunomodulator picropliv and ketoconazole effectively treated the *Leishmania donovani-*infected hamsters with improved cell-mediated immune responses (75). Miltefosine in combination with *L. braziliensis* antigens, saponin, and monophosphoryl lipid-A enhances CD4+ T cells in splenocytes producing IFN-γ and TNF-α and a reduction of IL-10 and anti-Leishmania circulating IgG in hamsters (76). A combination of miltefosine and recombinant cysteine proteinase from *Leishmania*, rldccys1, significantly reduced the parasite load in infected hamsters (77). Notably, IL-2 reduced *L. donovani* parasite burdens by 50% while IL-12 treatment reduced parasitic burdens by 47% and IFN-γ decreased them by 40%. Combination therapy of IFN-γ in addition to IL-12 enhanced the efficacy of a suboptimal dose of AmB against *L. donovani*. IL-10 and IL-27 are both suitable antileishmanial targets for neutralization in *L. donovani*-infected liver. In conclusion, both immunotherapy and immunochemotherapy could boost the efficacy of drugs (62, 132).

### Future prospective of immunotherapy and immunochemotherapy in VL elimination

Leishmaniasis is a severely neglected tropical disease with high mortality and morbidity rates especially in underdeveloped regions. The potential chemotherapeutic agents suffer from certain pitfalls such as parenteral administration, poor patient compliance, high dose, toxic side effects, and development of resistance (138). Regulation of certain co-stimulatory molecules, chemokines or chemokine receptor agonists, and costimulatory and cell signaling pathways has been known to reduce parasitic burden in *Leishmania* infection models. Therefore, focusing on the mechanism of host immune evasion by the parasite offers potential targets for immunotherapy. Such strategies aiming at enhancing the efficacy of chemotherapeutic agents with specific immunomodulators could serve as promising targets for immunochemotherapy (139). Moreover, immunochemotherapy can elicit an immune response clearing infection more efficaciously and providing more comeback trial possibility of recovery in patients. The current immuno-chemotherapies have shown a synergistic effect with antimonials which are known to show resistance in the Indian population. Therefore, future studies should be directed at the use of current first-line drugs such as AmB, and warrants further investigations as an immunochemotherapeutic. New research opportunities could advance the development of novel drug delivery systems encapsulating potential immunomodulators with and without drugs leading to the propagation of protective immunity (140). Nonetheless, the substantial cost of immunotherapy clearly necessitates the significance of new drug discovery platforms and pharmacological studies which are not only stable at tropical temperatures but can be administered in a patient-complaint manner. Although licensed vaccines are present, the scope for improvement remains undeterred which in future could lead to the development of safe and affordable vaccines (141).

### Current limitations of immunotherapy

A single dose of IL-27 or IL-10 monoclonal antibody enhanced macrophage activation, leading to an enhanced parasitic killing (56%). However, combination treatment with both anti-IL-27 and anti-IL-10 did not augment the immunotherapeutic antileishmanial effects (63). Combinations of immunomodulators reduce the parasite burden and at the same time accelerate self-cure. Although, much of the area is still straggling due to the absence of robust clinical studies (64). Furthermore, antibody neutralization of TGF-β augments IFN-γ production and hence enhances Th1-associated (IL-2) macrophage activation, nitric oxide production, and cytotoxic T lymphocyte proliferation, leading to the cure of VL independently of Th2-type cytokines (IL-4) (142). Furthermore, this hypothesis needs to be explored further to be used as a distinct possibility for VL treatment. Inhibition of IL-27 imposes negative regulates on IL-17 production which leads to disease exacerbation. Additionally, the inhibition of IL-10 contributes to the regression of VL (62). Treatment with IFN alone was only marginally effective, and conversely, it was more effective in combination with chemotherapy in VL patients (142). The current immunochemotherapy has shown a synergistic effect with antimonials which are known to show resistance in the Indian population. Treatment with antimonials alone did not show any difference among Indian patients. However, few patients were cured when treated with the most efficient immunochemotherapy. Therefore, future studies should be directed at the use of first-line drugs such as AmB, and warrants further investigations as an immunochemotherapeutic agent (140). A rudimentary understanding of detailed pathways and precise mechanisms underlying pathogenesis is still unclear. Translating experimental results into treatment strategies serves as a major hurdle due to the difference in immunohistopathologies of experimental models from humans. Furthermore, even though immunotherapy has given promising antileishmanial results without any severe toxic side effects, the immunomodulators might exacerbate an impervious immune response and pose a higher risk of sensitization along with the development of allergic reactions. Finally, the prophylactic investigation into these treatment strategies remains unresolved (143).

## Conclusion

The conventional and cost-effective therapeutic options for VL suffer from several limitations encompassing from being toxic to having adverse drug reactions (ADRs). Furthermore, the use of an effective lipid-based formulation (AmBisome) is confined mainly due to its high cost and instability in tropical conditions. To surmount these limitations, one best alternative approach could be an extensive understanding of pathogenesis and immunological events that ensue during VL infection, vital for the development of immunotherapeutic strategies against VL. Immunotherapy alone or its combination with conventional anti-leishmanial chemotherapeutic elixir (immunochemotherapy) could warrant an effective therapeutic option in future. However, such attempts need an explicit outlook toward the standardization procedures, and *in vivo* animal models could further help to understand the immunological footing and confer the possibility to translate it to clinical settings.

## Author contributions

All authors listed have made a substantial, direct, and intellectual contribution to the work and approved it for publication.
